# TGF-β signaling: critical nexus of fibrogenesis and cancer

**DOI:** 10.1186/s12967-024-05411-4

**Published:** 2024-06-26

**Authors:** Anna O. Giarratana, Conor M. Prendergast, Mary M. Salvatore, Kathleen M. Capaccione

**Affiliations:** 1 Northwell Health – Peconic Bay Medical Center, 1 Heroes Way, Riverhead, NY 11901 USA; 2 New York Medical College, Valhalla, NY 10595 USA; 3 Department of Radiology, Columbia University, New York, NY 11032 USA

**Keywords:** TGF-β, Cell signaling, Fibrosis, Cancer, Theranostics, Precision medicine

## Abstract

The transforming growth factor-beta (TGF-β) signaling pathway is a vital regulator of cell proliferation, differentiation, apoptosis, and extracellular matrix production. It functions through canonical SMAD-mediated processes and noncanonical pathways involving MAPK cascades, PI3K/AKT, Rho-like GTPases, and NF-κB signaling. This intricate signaling system is finely tuned by interactions between canonical and noncanonical pathways and plays key roles in both physiologic and pathologic conditions including tissue homeostasis, fibrosis, and cancer progression. TGF-β signaling is known to have paradoxical actions. Under normal physiologic conditions, TGF-β signaling promotes cell quiescence and apoptosis, acting as a tumor suppressor. In contrast, in pathological states such as inflammation and cancer, it triggers processes that facilitate cancer progression and tissue remodeling, thus promoting tumor development and fibrosis. Here, we detail the role that TGF-β plays in cancer and fibrosis and highlight the potential for future theranostics targeting this pathway.

## Background

### The role of TGF-β signaling

In cellular biology, transforming growth factor-beta (TGF-β) signaling emerges as a crucial modulator of cell fate, orchestrating processes from cell proliferation to apoptosis. In this review, we aim to dissect the dual roles of TGF-β signaling in both fibrosis and cancer, elucidating its paradoxical behavior across different cellular contexts. We hope to integrate both established knowledge and recent findings to enhance our understanding of the full role that TGF-β signaling plays. In addition to discussing what has been discovered about TGF-β signaling, we also aim to identify potential therapeutic targets within these pathways. These targets may play a significant role in the future of medical therapies for both cancer and fibrosis. The overarching aim of our review is to summarize the existing knowledge of TGF-β signaling and to clarify how this understanding guides our identification of potential therapeutic targets.

### TGF-β signal transduction

The TGF-β signaling pathway is a crucial regulator of numerous cellular processes including proliferation, differentiation, apoptosis, and extracellular matrix production [1]. In humans, the TGF-β superfamily is comprised of over 30 related cytokines, which act in a context-dependent manner [2]. These signal transduction pathways play an important role in both normal physiology and pathophysiology with actions in tissue homeostasis, fibrosis, and cancer progression [3].

The canonical TGF-β signaling cascade is initiated by the binding of TGF-β ligands to cytoplasmic serine/threonine kinase receptors to form a heteromeric complex. TGF-β binds the type I receptor (TβRI), which then recruits the type II (TβRII) receptor via phosphorylation. This activation initiates a series of intracellular signaling events, primarily mediated by the small mothers against decapentaplegic (SMAD) family of proteins [4]. Receptor SMAD (R-SMAD) is phosphorylated by the activated TβRI, which in turn promotes the binding of common mediator SMAD4 (co-SMAD) to the complex. These SMAD complexes subsequently translocate to the nucleus, where they interface with transcription factors, coactivators, and corepressors to modulate the transcription of target genes [5, 6]. Canonical TGF-β signaling is subject to regulation by inhibitory SMADs (I-SMADs), of which SMAD6 and SMAD7 often play an important role by interfering with either receptor activation or SMAD complex formation [7].

**Fig. 1:**
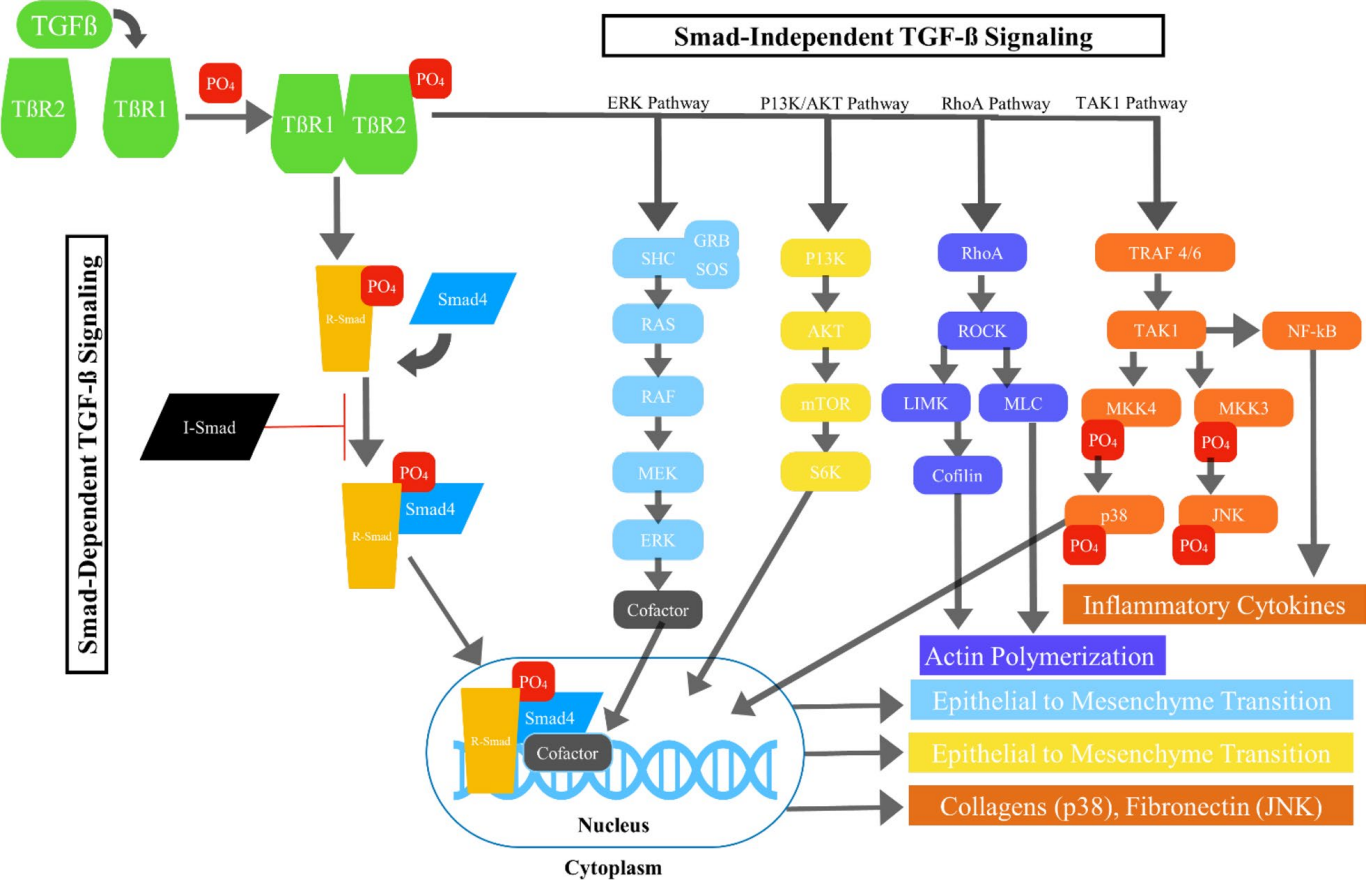
Graphical illustration of both the canonical SMAD and non-canonical non-SMAD TGF-β signaling pathways. TGF-ß has multiple pathways with which to influence fibrosis, ultimately leading to the induction of inflammatory cytokines and polymerization of actin, as well transcription factors for expression of extracellular matrix proteins and differentiation to mesenchyme

In addition to the canonical TGF-β signaling pathway which signals through SMAD proteins, there are non-canonical, non-SMAD TGF-β signaling pathways (Fig. 1). TGF-β signaling effectively crosses over from the canonical signaling pathways to the non-canonical signaling pathways via mediators such as Mitogen-activated protein kinase (MAPK), phosphoinositide 3-kinase (PI3K)/protein kinase B (AKT), Rho-like guanosine triphosphatases (Rho-like GTPases), and nuclear factor-kappa B (NF-κB) in a context-specific manner, often influenced by mechanisms such as receptor complex composition, post-translational modifications, and crosstalk with other signaling pathways. This shift is often dictated by the cellular context, including cell type, microenvironmental factors, and the presence of specific adaptor proteins, leading to diverse biological outcomes in response to TGF-β stimulation, to be discussed in further detail below [1, 3, 8, 9].

Non-canonical TGF-β signaling includes pathways such as the MAPK cascades, (PI3K)/AKT, Rho-like GTPases, and (NF-κB) signaling pathways [10]. The activation of non-SMAD pathways often occurs downstream of TβRI or through the recruitment of adaptor proteins, such as TNF receptor-associated factor (TRAF6 and TRAF4, which facilitate the formation of signaling complexes at the receptor level [11, 12]. Importantly, interactions between the canonical and noncanonical TGF-β signaling pathways are vital for effective fine-tuning the cellular output of TGF-β stimulation.

### Clinical manifestations of TGF-β signaling in normal physiological circumstances

The downstream effects of TGF-β signaling are both cell type and context-dependent and can often cause antagonizing effects. In normal physiological circumstances TGF-β signaling often promotes cellular quiescence and apoptosis, serving as a potent tumor suppressor [13]. TGF-β signaling can upregulate Cyclin-dependent kinases (CDK) inhibitors, such as p21 and p15, stimulating cell cycle arrest by promoting cellular quiescence [14]. At the same time, TGF-β signaling can downregulate positive cell cycle regulators, such as cyclin D1 [15]. These actions inhibit cell cycle progression and promote cell cycle arrest and quiescence. In addition, TGF-β signaling can both upregulate pro-apoptotic genes, such as Bcl-2 Interacting Mediator of cell death (*Bim*) and B-cell lymphoma 2 modifying factor (*BMF*), and downregulate anti-apoptotic genes, such as B-cell lymphoma 2 (*BCL2*) and B-cell lymphoma-extra-large (*BCL-xL*) [16, 17]. These actions contribute to the role of TGF-β signaling in inducing apoptosis. Taken together, these actions highlight the role that TGF-β signaling plays in tumor suppression in normal, non-pathological conditions.

### Clinical manifestations of TGF-β signaling in pathological conditions

In pathological conditions, TGF-β signaling can seemingly have the exact opposite effect. In the context of cancer at certain stages or other pathological conditions where there is an altered cellular microenvironment, TGF-β signaling can switch to playing the part of a tumor promoter [18]. In these conditions, TGF-β signaling upregulates mesenchymal markers, such as N-cadherin and vimentin. At the same time, it can downregulate epithelial markers such as E-cadherin and cytokeratins. Taken together, these actions promote the epithelial-to-mesenchymal transition (EMT), an important factor in the ability of cancer to migrate, invade, and metastasize [17, 19]. TGF-β signaling increases extracellular matrix (ECM) components like fibronectin, collagen, matrix metalloproteinases (MMPs), and tissue inhibitors of metalloproteinases (TIMPs) in certain microenvironments, leading to ECM degradation, tissue remodeling, and fibrosis [9, 20]. While TGF-β signaling can seemingly have opposing actions, there is another way to contextualize the downstream effects on TGF-β signaling. In fibrosis, TGF-β is a key mediator of fibroblast activation and ECM production, leading to tissue scarring and organ dysfunction. This fibrotic process creates a microenvironment that can be conducive to tumor development and progression, suggesting a potential pro-carcinogenic role for TGF-β in fibrotic tissues [9]. These actions are mediated by the TGF-β signaling transitions from canonical to non-canonical pathways and are dependent on factors like receptor composition, post-translational modifications, and cell-specific contexts [1, 3, 8, 9].

### TGF- β as a meditator of cell motility and EMT

TGF-β is a crucial mediator of cell motility and EMT, processes crucial to wound healing and cancer metastasis. TGF-β signaling induces epithelial cells to lose their polarity and cell–cell adhesion. These cells then acquire a mesenchymal phenotype, with enhanced migratory and invasive capabilities. This transformation is characterized by downregulation of epithelial markers and upregulation of mesenchymal markers. Studies have shown that these changes are medicated through Smad proteins, MAPKs, and the Rho/Rac1 family of small GTPases [21, 22]. In particular, studies have shown that there is a synergistic interaction between Rho and Rac1 enhancing EMT and cell motility [23]. This shift facilitates tissue remodeling and is implicated in the development of fibrosis as well as the progression and metastasis of various cancers.

### TGF- β as a meditator of cancer stemness

TGF-β also is a pivotal regulator in maintaining and promoting cancer stemness, a property of cancer cells that contributes to tumor initiation, progression, and drug resistance. When cancer cells develop stemness, they have the ability to self-renew, differentiate, and are responsible for driving tumor growth and metastasis. They often are more resistant to conventional therapies like chemotherapy and radiation due to their enhanced deoxyribonucleic acid (DNA) repair capabilities and a slow-dividing nature [24]. Their ability to self-renew leads to tumor regrowth and metastasis even after initial treatments appear successful, while their slow cell cycle progression means they are not as affected by treatments that target rapidly dividing cells [25]. Studies have shown that when TGF-β1 is secreted, cancer cell stemness is maintained, and the cancer becomes resistant to treatment. In particular this was shown in an esophageal squamous cell carcinoma model, where cisplatin treatment was trialed [26]. In addition, the activation of TGF-β signaling pathways leads to similar results; in a study where cancer upregulated gene 2 (CUG2) was activated, TGF-β signaling pathways were activated, which led to stemness phenotypes in cancer cells. However, the suppression of Smad and non-Smad TGF-β signaling pathways prevented CUG2 from inducing these stemness-like phenotypes [27]. The time course has also been shown to be important, with one study showing in an in vitro cell model, that in more prolonged, chronic TGF-β exposure, that the mechanistic target of rapamycin (mTOR) signaling pathway was activated, inducing both EMT and cancer stemness, which mimicks the actions of a carcinoma in vivo [28]. Epigenetics are thought to play an important role in the activation of these pathways. One study showed that treatment of human cell lines with TGF-β results in epigenetic activation through promoter demethylation of genes such as slug and urokinase plasminogen activator receptor (CD87), resulting in increased cancer stemness [29]. While another study showed that in a pancreatic ductal adenocarcinoma cell model, TGF-β signaling via SMAD2/3 induced microRNA (miR) 100 host gene long noncoding RNA (lncRNA), which led to the expression of miR-100 and miR-125b, mediators of cancer stemness [30]. TGF-β modulation of cancer stemness is a key factor in the development of more aggressive and treatment resistant cancer cell phenotypes.

### TGF- β as a meditator of drug resistance

TGF-β signaling plays a significant role in mediating drug resistance in various cancers via actions on the expression of drug efflux transporters, alteration of cell cycle checkpoints, and modulation of apoptosis pathways, all which can reduce the efficacy of anticancer drugs [31–35]. TGF-β signaling has been linked to the development of resistance to chemotherapy and targeted therapies. Due to its strong association with resistance to immune checkpoint inhibitors, hyperactive TGF-β signaling has been proposed as a potential biomarker for cancer treatment resistance, particularly in checkpoint inhibitor therapies [34]. In particular, one study has shown that the increased mitogenic signaling in cancer states favors alternative splicing events that enables TGF-β-activated Smad3 to collaborate with poly(RC) binding protein 1, which promotes the expression of protein variants crucial for EMT [36]. Another study found that TGF-β signaling is a key factor in the maintenance of stem cell homeostasis, and thereby plays a role in regulating the stemness of cancer cells, conferring resistance to treatment approaches. These studies highlight the role of TGF-β signaling in the complex tumor microenvironment, focusing on its impact on EMT and cancer stemness, and how activating TGF-β signaling can lead to treatment resistance.

## Main text

### The dual role of TGF-β signaling

Central to our discussion here is the critical nexus between fibrogenesis and cancer. Both of these are mediated by TGF-β signaling in a context-dependent manner. Fibrogenesis often precedes cancer progression, with TGF-β acting as a key facilitator in this pathway by remodeling the extracellular matrix, and thereby creating a conducive microenvironment for both tumor growth and metastasis.

In fibrotic tissues, TGF-β drives not only enhanced fibroblast activation but it also modulates immune surveillance. Once these pathways are activated, the result is neoplastic transformation and cancer cell survival. The intersection of these pathways underscores the pivotal role of TGF-β signaling in the transition from a state of tissue repair to a state that is conducive to tumor growth. This dual functionality of TGF-β signaling highlights the complexity of targeting TGF-β in therapeutic strategies. The inhibition of TGF-β signaling can impact both fibrosis and cancer pathways, which may be at odds with the therapeutic goal.

### TGF-β signaling in fibrosis

TGF-β signaling plays an important role in fibrosis. Fibrosis is a process by which there is excessive deposition of (ECM) proteins leading to organ dysfunction and resulting in significant morbidity and mortality for patients [3, 37]. TGF-β signaling is a vital component of the fibrogenic process. The TGF-β signaling pathways, both the canonical SMAD signaling pathway as well as the non-canonical pathways, activate mechanisms ultimately leading to increased fibrosis. This occurs in a few different ways, with TGF-β signaling acting on many different pathways responsible for ECM production and maintenance [38]. TGF-β signaling induces the activation and differentiation of fibroblasts into myofibroblasts via actions on TβRII and TβRI, leading to the phosphorylation of SMAD2 and SMAD3, which form complexes with SMAD4 and translocate into the nucleus to regulate the transcription of fibrosis-related genes [5, 6]. Myofibroblasts are the principal cells that are responsible for ECM production, leading first to ECM accumulation and then to tissue scarring and organ fibrosis [39]. This increase in myofibroblasts results in the excessive production and deposition of ECM. Activation of these signaling pathways promotes the synthesis of the elements of ECM, such as collagen, fibronectin, and proteoglycans. As discussed above, this process is often associated with EMT, a phenomenon where epithelial cells acquire mesenchymal, fibroblast-like properties, enhancing their mobility and capacity for ECM production, and playing a crucial role in both fibrosis and cancer metastasis [40].

In fibrosis, TGF-β is overexpressed, and the context that this occurs in has an effect on the downstream signaling pathways. As discussed previously, the canonical signaling pathway has a role in this process. For example, in lung fibrosis, researchers have shown that the fate of alveolar epithelial cells in response to TGF-β signaling is dependent upon the activation of focal adhesion kinase (FAK). When SMAD3 and FAK are activated, TGF-β signaling induces EMT. On the other hand, when FAK is inhibited, cells are sent down an apoptotic pathway [41]. Other researchers have shown that TGF-β activates other non-canonical pathways as well. The activation of the P38 MAPK pathway is associated with the induction of α-smooth muscle actin (α-SMA) in myofibroblasts, a key indicator of fibroblast activation. Researchers have demonstrated that by targeting miR-375 in a human lung fibroblast model, they can inhibit this pathway and modulate its signaling [42]. Another group showed that TGF-β can induce EMT via the activation of the Erk signaling pathway, and that inhibition of this signaling pathway blocks EMT in a mouse model [43].

While TGF-β is primarily associated with the promotion of ECM production and fibrosis, it can also be involved in ECM remodeling and degradation under certain conditions. In particular, MMPs play a crucial role in ECM remodeling by breaking down various ECM components, while TIMPs regulate this process by inhibiting MMP activity, thus maintaining a balance essential for normal tissue function and repair. In some contexts, TGF-β can induce the expression of specific MMPs, such as MMP2 and MMP9, which are capable of degrading components of the ECM [44–46]. However, this effect is often counterbalanced by the simultaneous induction of TIMPs, which can lead to a net accumulation of ECM in the fibrotic process [47–50]. From this, it can be seen that TGF-β signaling is indeed context-dependent; similar to the role that TGF-β signaling plays in cancer with both pro- and anti-apoptotic pathway activation, in fibrosis, TGF-β signaling can result in the activation of pro- and anti-fibrotic pathways influenced by the cellular environment, the presence of other cytokines and growth factors, and the specific type of tissue involved [9]. We have chosen to focus on a more in-depth discussion on the role of TGF- β signaling in fibrosis in the lung, breast, and liver due to the significant prevalence and impact of fibrotic diseases in these organs, which are often linked to cancer progression and highlight critical areas for therapeutic intervention.

#### TGF-β signaling in lung fibrosis

Pulmonary fibrosis is a debilitating condition characterized by progressive scarring of lung tissue. Idiopathic pulmonary fibrosis (IPF) is one of the most common clinical manifestations of this process [51]. A recent review estimated that the rates of IPF are increasing, with an incidence rate of 3–9 cases per 100,000 people per year in Europe and North America [52]. Once diagnosed with IPF, the median survival is 3–5 years, similar to lung cancer [39]. Importantly, TGF-β signaling has been implicated in the pathogenesis of IPF. Previous work in rat models of IPF showed alveolar epithelial cells that overexpressed the biologically active TGF-β1 were characterized by lesions similar to what are seen in IPF, with hyperplasia, increased number of fibroblasts, and interstitial thickening [53, 54]. The role of increased TGF-β signaling causing IPF is thought to be due to a number of different mechanisms downstream of the initial signaling [39, 55], including differentiation of fibroblasts into myofibroblasts, resulting in excessive production and deposition of ECM components. TGF-β also is important in the balance of ECM synthesis and degradation, with overexpression of TGF-β signaling tipping the scale towards accumulation of fibrotic tissue. SMAD and non-SMAD signaling pathways confer resistance to anoikic-mediated cell death, an important process that is impaired in IPF, through which a cell is stimulated to undergo apoptosis after detachment from the correct ECM [56]. Currently, there are two Food and Drug Administration (FDA) approved treatments for IPF; pirfenidone and nintedanib [55]. Researchers have shown in a bleomycin pulmonary fibrosis mouse model, that one of the mechanisms of action of the drug pirfenidone is the suppression of the overactive TGF-β1/Smad2/3 signaling pathways in IPF [57]. These results highlight the importance of TGF-β signaling in lung fibrosis and the efficacy of targeting this signaling pathway as an antifibrotic strategy (Table 1).

**Table 1:** The key cell signaling players in fibrosis, their mechanisms, and currently developed inhibiting drugs

Molecule	Mechanism	Pharmacological inhibitors	Source
Transforming Growth Factor ß (TGFß)	Proliferation of fibroblasts and macrophages via SMAD pathway	Vactoserib Luspateracept Bintrafusp	[1, 69–71]
Interleukin-13 (IL-13)	Induces myofibroblast differentiation and TGFß1 production via STAT6	Tralokinumab Lebrikizumab Eblaskimab	[72, 73]
Nuclear Factor kappa B (NF-κB)	Promotes expression of inflammatory cytokines	Statins/Ezetimibe Decoy Oligonucleotides	NCT01424891 NCT00125333
Vascular Endothelial Growth Factor D (VEGF-D)	Stimulates angiogenesis and lymphogenesis via tyrosine kinase pathway; down regulated by TGFß1	Pazopanib Sunitunib Bevacizumab	[75, 76]
Platelet Derived Growth Factor (PDGF)	Mitogen for mesenchymal cells, including fibroblasts	Imatinib Linifanib Nilotinib	[77]
c-Jun N-terminal Kinase (JNK)	Modulation of apoptosis, cell differentiation, and cytokine and fibronectin expression	CC-90001	NCT00125333

Several of these listed, including TFG-ß, IL-13, PDGF, and JNK are closely involved with cell differentiation into mesenchyme. Inhibition of these is believed to have clinical significance in controlling fibrosis

#### TGF-β signaling in breast density

Increased breast density is associated with a 1.6-fold increased risk of breast cancer. Increased breast density is usually assessed on mammography, with a Breast Imaging Reporting and Data System (BI-RADS) rating of D signifying extremely dense breast tissue [58]. Breast tissue that has a BI-RADS rating of D is characterized by a higher proportion of stromal and epithelial tissue relative to fatty tissue [59]. Of note, TGF-β signaling is thought to contribute to increased breast density through its downstream signaling effects [60]. Given that dense breast tissue has a higher proportion of stromal tissue, and that fibroblasts are the most common cell type within the mammillary stroma, it is logical that increased TGF-β signaling leads to the sustained activation of fibroblasts, resulting in the eventual excessive deposition of collagen and other ECM products and development of fibrosis. The increase in fibrosis in breast tissue increases the risk for eventual tumor development, as the altered ECM and stromal environment can create a niche that supports tumor cell survival, proliferation, and invasion, thereby facilitating the initiation and progression of breast cancer. One study used a bi-transgenic tumor mouse model to investigate the role that increased stromal collagen has in tumor formation, and found that in collagen dense micro-environments, tumors were more likely to both form and metastasize [61]. Another study found that a high-density ECM utilizes TGF-β signaling to reduce T cell proliferation and alter their differentiation, favoring regulatory over cytotoxic T cells, which impairs the immune function to kill cancer cells [62]. Other studies have directly linked the overexpression of TGF-β–β3 integrin signaling with metastasis in a mouse model of breast cancer [63]. These results highlight the importance of further study into the role that TGF-β signaling plays in breast density, and the ways it may be targeted to prevent progression to malignancy.

#### TGF-β signaling in cirrhosis

Cirrhosis is the clinical outcome when the liver has been inflamed for a prolonged period of time. As a result of inflammation, the healthy liver begins to transform into fibrotic tissue. This fibrosis of the liver can lead to eventual liver failure, a clinical problem with significant morbidity and mortality [64]. TGF-β signaling plays a key role in the pathogenesis of liver cirrhosis [65]. In the liver, activation of the hepatic stellate cells leads to the differentiation of myofibroblasts, which can result in ECM synthesis and eventual fibrosis. TGF-β signaling is thought to play a role in this activation [66]. Recent work has attempted to target this pathway by utilizing molecules that inhibit TGF-β/Smad in a mouse model of liver fibrosis, showing that disease progression is slowed by these actions [67]. Additionally, work investigating the clinical potential of Galunisertib, a TGF-β receptor type I kinase inhibitor, has showed that treatment with the drug inhibits the phosphorylation of SMAD2, and reduced fibrosis related markers in a human ex vivo model of liver fibrosis [68].

### TGF-β signaling in cancer

In addition to the role that in plays in fibrosis, TGF-β signaling plays a complex and context-dependent role in cancer, switching between pro- and anti-carcinogenic effects [81]. Prior to the transformation of a cell to a cancer cell, and even up to the early stages of cancer, TGF-β signaling typically exerts tumor-suppressive effects by inhibiting cell proliferation, inducing apoptosis, and maintaining genomic stability. These actions are thought to be mediated through the TGF-β-Smad-Snail signaling axis [82]. However, as the tumor microenvironment evolves, a critical shift occurs in TGF-β signaling to a tumor-promoting role. Factors such as hypoxia, immune cell infiltration, and changes in ECM composition contribute to this transition. In the tumor microenvironment, chronic inflammation and the presence of cytokines can lead to the activation of alternative non-canonical signaling pathways, such MAPK, PI3K/AKT, Erk1/2, Rho-like pathways, c-Jun N-terminal kinase (JNK), and p38/MAPK [3]. These interactions result in reduced tumor suppressive SMAD signaling, and as a result, TGF-β signaling transitions to a tumor-promoting role.

This shift in TGF-β signaling facilitates cancer cell invasion, metastasis, and EMT [18]. In addition, TGF-β signaling also contributes to the formation of a pro-tumorigenic microenvironment by stimulating angiogenesis, enhancing the production of extracellular matrix components, and promoting matrix remodeling [17, 19]. Further, TGF-β signaling supports cancer immune evasion by suppressing the activation and proliferation of immune cells and by fostering the differentiation of immunosuppressive cell populations, such as regulatory T cells (Tregs) and myeloid-derived suppressor cells (MDSCs) [83, 84]. Through all these mechanisms, TGF-β signaling acts as a cancer promotor, and facilitates cancer resistance to therapy across various cancer subtypes. We have chosen to focus on TGF-β signaling in pancreatic cancer, lung cancer, breast cancer, and glioma because of the high incidence and mortality rates associated with these cancers. Understanding their distinct biological mechanisms and clinical challenges will provide crucial insights into devising effective treatment strategies and enhancing patient outcomes (Table 2).

**Table 2:** Main molecular players in cancer via TGF-ß pathway

Protein	Effects	Common cancers	Source
Cyclin-dependent kinase 4 inhibitor B (P15)	Induced by TGF-ß; complexes CDK4/6 to stop activation of Cyclin D, arresting cell in G1	Bladder, nasopharyngeal, kidney, lung cancer	[14, 15, 114, 121]
Cyclin-dependent kinase inhibitor 1 (P21)	Activated by p53 in response to DNA damage; complexes CDK2 and PCNA to induce gene repair	Colorectal carcinoma, breast cancer, tonsillar cancer, squamous cell carcinoma, gastric and pancreatic cancers	[14, 87, 88, 105, 115, 122, 123]
SRY-box 9 (Sox9)	Transcription factor for embryonic and stem cell differentiation; functions as a tumor suppressor and oncogene	Bladder, colorectal carcinoma, melanoma, NSCLC	[98, 119, 120, 124, 125]
Cellular Abelson (c-Abl)	Proto-oncogene encoding tyrosine kinase receptors for cell differentiation and division	*ABL1 and ABL2:* Lung adenocarcinoma, colon adenocarcinoma, melanoma, breast invasive ductal carcinoma *BCR-ABL:* Chronic myeloid leukemia	[105, 126, 127]
Human epidermal growth factor 2 (HER2)	Tyrosine kinase receptor, activating MAPK, PI3, PKC, and Phospholipase C pathways	Breast, gastrointestinal, esophageal	[108, 128]
Epidermal growth factor receptor (EGFR)	Tyrosine kinase receptor, activating MAPK, Akt, and JNK pathways for DNA and cell proliferation	Glioblastoma, breast, prostate, NSCLC, ovarian	[95, 108, 110, 129, 130]
Kirsten Rat Sarcoma Viral Oncogene Homolog (Kras)	GTPase intermediate of the RAS/MAPK pathway for cell growth	Pancreatic, NSCLC, colon	[95, 96, 131]
Small mother against decapentaplegic (Smad)	Transduction factors activated by TGF-ß, modulating chromatin activation as tumor suppressors	*Smad2:* Colon, head, neck *Smad4:* Breast, colon, esophagus, pancreas, ovary, glioma	[3, 87, 116, 117, 132, 133]

This table delineates several key proteins, including P21, Sox9, c-Abl, HER2, EGFR, Kras, and Smad, which are induced or activated through the TGF-ß signaling. Detailed here is the protein’s role in cell cycle regulation, DNA damage response, cell differentiation, and tumor suppression or oncogenesis, as well as their association with common cancers

#### TGF-β signaling in pancreatic cancer

Pancreatic ductal adenocarcinoma (PDAC) is a particularly lethal cancer, with 5 year survival rates of 2–9%, and no effective treatments currently [85]. As the case in general for TGF-β signaling, it plays a complex dual role [86]. In pre-cancerous states, TGF-β signaling through the Smad signaling pathway has antiproliferative effects. However, in research that has investigated two pancreatic carcinoma cell lines, PT45 and Panc-1, that are resistant to the normal anti-proliferative effects of TGF-β signaling, it was found that the Smad signaling pathway activation is decreased. This was shown in comparison to epithelial cell lines, that are sensitive to the normal anti-proliferative effects of TGF-β signaling. In addition, they found that expression of the Cyclin-dependent kinase (CDK) inhibitor, p21, is decreased in the resistant pancreatic cancer cell lines compared to the sensitive epithelial cell lines [87]. Given the function of p21 which is required for the antiproliferative effects TGF-β these results indicate that over time, pancreatic cancer cells are able to become resistant to the early antiproliferative effects of TGF-β signaling. Another group found that in human pancreatic cancer cell lines, that loss of deleted in pancreatic cancer 4 (DPC4), homologous to Smad4, expression disrupts TGF-β signaling, reducing the ability to inhibit cell growth and regulate target genes like *p21waf1*, which may contribute to unchecked cell proliferation in pancreatic cancer [88].

In humans, as pancreatic cancer cells start to lose this responsiveness to TGF-β Smad signaling, the levels of TGF-β isoforms and mRNA expression in pancreatic cancer cells start to increase. This increase in TGF-β in pancreatic cancer cells has been correlated with shorter survival times, making it an important prognostic factor in this disease [89]. Given these results, it is of interest to researchers to discover if inhibitors of TGF-β signaling could be an effective targeted therapy for pancreatic cancer. There are currently a handful of clinical trials in stages I-II investigating the effect of TGF-β inhibitors in PDAC patients [90].

#### TGF-β signaling in lung cancer

Lung cancer is the number one cause of death from cancer in North America [91, 92]. Because of this, efforts have been made to further characterize the processes that occur in lung cancer, to better understand, diagnose, and treat the condition. TGF-β signaling is thought to play a significant role in lung cancer. The majority of lung cancer cases are non-small cell lung cancer (NSCLC), a category which includes adenocarcinoma and squamous cell carcinoma. On the other hand, small cell lung cancer (SCLC) only represents 10–15% of lung cancers but is highly aggressive when it does occur [93].

In NSCLC, researchers have shown that the level of TGF-β1 protein levels within tumor tissue is significantly associated with lymph node metastasis, advanced disease stages, and increased micro-vessel density, indicating its role in tumor angiogenesis and progression. In particular, they found that in patients with adenocarcinoma, high TGF-β1 protein levels correlate with poorer prognosis [94]. In NSCLC, in particular in adenocarcinoma, mutations are common in epidermal growth factor receptor (EGFR) and Kirsten rat sarcoma viral oncogene homolog (KRAS). Given the important role these factors play in the EMT, this action can promote the change from tumor-suppressive to tumor-promoting [95]. In a NSCLC cell line, researchers investigated the role that the KRAS mutations, in particular the G12V mutation, have on the TGF-β signaling pathways and outcomes. They found that the KRAS^G12V^ mutation increases programmed death-ligand 1 (PD-L1) expression, promoting immune escape via the TGF-β EMT signaling pathway [96]. Another group investigating the role that cell cycle proteins play in TGF-β signaling in NSCLC, found that the concordant expression of TGF-β1 and p21waf1/cip1 was correlated with improved disease-free survival independent of grade, stage, or p53 status [97]. It has also been shown that sry-related HMG-box (Sox)9 plays a crucial role in cancer progression in NSCLC, especially in adenocarcinoma, by inducing a mesenchymal phenotype, promoting cell motility and invasion, and interacting with multiple signaling pathways including neurogenic locus notch homolog protein (Notch), TGF-β, NF-κB, Bone morphogenetic protein (BMP), EGFR, and Wingless/Integrated (Wnt)/β-catenin [98].

In SCLC, the tissues that are used for analysis are often very heterogenous [99, 100]. Most SCLC cell lines used in research are unresponsive to the inhibitory effects of TGF-β signaling due to a lack of the TGFβR-II, leading to cancer cell immune resistance [100]. However, other SCLC cell lines do express TGFβR-II. The role of the heterogeneous TGF-β signaling in SCLC is being investigated, and further study will be required to parse out the role that TGF-β signaling plays in SCLC [93]. Interestingly, in patients who have pulmonary fibrosis, squamous cell cancer is the most common cancer to develop [101].

#### TGF-β signaling in breast cancer

Breast cancer is one of the most common cancers diagnosed in women and is the leading cause of cancer mortality in women [102]. Recent figures state that there were 2.26 million global cases of breast cancer in 2020 [103]. As in other cancers, TGF-β signaling plays a dual role in breast cancer, functioning as both a tumor suppressor and promoter depending on the cellular context and disease stage.

In breast cancer, TGF-β mediates cell cycle arrest by regulating cyclin-dependent kinases and their inhibitors, but altered responsiveness to TGF-β, often due to dysregulation of cell cycle effectors or mutations in TGF-β signaling components, contributes to breast cancer progression [104]. Similar to what has been found in pancreatic cancer, p21 seems to play an important role in tumorigenicity and TGF-β signaling. In a study using triple-negative breast cancer cells, researchers found that c-Abl activity leads to tumor suppression by upregulating p21Waf1/Cip1, which induces senescence and diminishes cancer stem cells, a mechanism crucial for reducing tumorigenesis and metastatic progression. However, in cells deficit in p21, their tumorigenicity was partially restored [105].

Another group of researchers studying the role of TGF-β and the cell cycle have investigated TGF-β signaling in the MCF-7 human breast cancer adenocarcinoma cell line. They found that TGF-β1 inhibits the proliferation of MCF-7 human breast adenocarcinoma cells by inducing cell cycle arrest at the growth 1 (G1) phase. In MCF-7 cells that lack the type II TGF-β receptor, this inhibitory effect is absent, suggesting that TGF-β1's action depends on this receptor to downregulate CDK2 kinase activity. Their findings suggest that TGF-β1 inhibits MCF-7 cell proliferation by downregulating CDK2 kinase activity via a type II receptor, while increasing nuclear accumulation of Cyclin-dependent kinase inhibitor 1A (p21WAF1)/Cyclin-dependent kinase interacting protein 1 (CIP1), all without changing CDK or cyclin expression CDKCDK [106].

In addition to the effects on the cell cycle, in breast cancer, TGF-β signaling has been found to be an important factor in cancer invasion and metastasis [107]. Researchers have shown in a mouse model that human epidermal growth factor receptor 2 (HER2) modulates TGF-β signaling through the AKT-mediated phosphorylation of Smad3, promoting cell migration and EMT of breast cancer cells [108]. In a human breast cancer cell model, one recent study found that there was a positive correlation between the expression of TGF-β and EGFR, an important growth factor known to be overexpressed in breast cancer. They found that treatment of the cells with TGF-β resulted in increased EGFR expression through both canonical Smad3 signaling and non-canonical extracellular signal-regulated kinase (ERK)/Specificity protein 1 (Sp1) signaling pathways. Another study found that TGF-β-induced EMT in a breast cancer cell model alters the response to EGF, leading to increased invasiveness and metastatic potential, which is characterized by changes in cell morphology, EGFR activation, and dependency on FAK [110].

These studies highlight the important role that TGF-β signaling plays in breast cancer cell invasion and metastasis when its function switches from antiproliferative to proliferative. Given the role of TGF-β signaling in breast cancer, researchers are now investigating it as a target for treatment in breast cancer [109, 111].

#### TGF-β signaling in glioma

Gliomas are the most common type of primary brain tumor, with significant morbidity and mortality [112]. Grade 4 glioma, known as glioblastoma, is the most frequent primary malignant brain tumor. Once diagnosed with glioblastoma, the median survival time is less than 2 years [113]. As with other cancers, TGF-β signaling plays a critical role in glioma development and progression through its multifaceted effects on tumor growth, invasion, angiogenesis, and immune evasion. Given the high degree of invasiveness and ability to metastasize, researchers have been interested in studying the role that TGF-β signaling plays in this cancer.

Researchers have been interested in studying the role of cell cycle proteins downstream of TGF-β signaling in glioma. One group used human glioma cell lines and found that TGF-β inhibits the proliferation of normal astrocytes by upregulating the CDK inhibitor p15(INK4B) and inducing G1 cell cycle arrest. In high-grade human gliomas, often with p15(INK4B) gene deletions, there were varied responses to TGF-β signaling, ranging from mild growth inhibition to stimulation, implicating the loss of p15(INK4B) in the reduced sensitivity of gliomas to TGF-beta’s growth-inhibitory effects [114]. Another group utilized decorin, a small leucine-rich proteoglycan, that interacts with TGF-β and p21, playing a significant role in carcinogenesis and tumor progression. The molecule binds to TGF-β, inhibiting its immunosuppressive effects and promoting the regression of rat C6 gliomas by reversing TGF-β induced immunosuppression. Although decorin has been shown to induce p21 expression and cell cycle arrest in some cancers, this effect is not observed in glioma cells, indicating that perhaps gliomas have diverse TGF-β signaling relative to other cancers [115].

TGF-β signaling through the SMAD signaling pathway has correlated with poor prognosis in patients with glioma [116, 117]. One study analyzed primary cultured patient-derived glioma cells and human glioma biopsies and found that TGF-β-SMAD signaling was correlated with more aggressive and proliferative gliomas. Using cell culture, they found that activation of this signaling pathway is dependent of epigenetic control; unmethylated platelet derived growth factor subunit B (*PDGF-B*) gene induced proliferation compared to methylated *PDGF-B* [118]. Another group used human cell and tissue models to study the downstream effects of TGF-β signaling in glioma. They found that TGF-β signaling prevents the degradation of the Sry-related high mobility group box (Sox) family transcription factor Sox9. This allows Sox9 to function unchecked, leading to increased invasiveness of glioma cells. They found that the upregulation of Sox9 due to TGF-β signaling was correlated with poor clinical prognosis in patients with glioma [119]. Treatments that target TGF-β signaling may be effective in multiple types of cancer, including pancreatic cancer, breast cancer, NSCLC, and glioma, more rigorous investigation in both preclinical and clinical settings is needed. These will be further discussed in the following sections of this review article [120].

### Evaluating and targeting TGF-β signaling

The most widely utilized methods of determining TGF-β signaling are in vitro methods that use patient samples. By using methods such as polymerase chain reaction, immunohistochemistry, immunofluorescence, and Western blot, the expression level of TGF-β ligands, receptors, or downstream targets in patient samples can serve as surrogate markers for TGF-β signaling activity. These measurements can potentially help identify patients who might benefit from anti-TGF-β therapies, monitor treatment response, or predict disease prognosis [32]. As research progresses, many groups are making efforts to study TGF-β signaling in vivo*.*

Recent advancements have expanded the in vivo study of TGF-β signaling to provide more comprehensive insights into its role in both physiological states and pathological disease. Mouse models and xenografts are frequently employed to replicate the complex tumor microenvironment and better understand the dynamic interactions between TGF-β signaling and cancer progression [134–136]. In addition, non-invasive imaging techniques, such as positron emission tomography (PET) and magnetic resonance imaging (MRI), have become increasingly valuable tools for tracking TGF-β signaling changes in pre-clinical animal models, offering crucial data on treatment efficacy and tumor behavior [137–141]. By combining in vitro and in vivo methods, the hope is that researchers will be able to further develop and refine their diagnostic and therapeutic strategies, ensuring more precise targeting of TGF-β pathways leading to improved patient outcomes in the long-term.

#### Therapies targeting TGF-β signaling

TGF-β signaling can have many profoundly different context-specific effects. Of note, increased TGF-β signaling can lead to both cell cycle arrest and apoptosis, or paradoxically, to proliferation and differentiation. In this way, it often acts as a tumor suppressor in healthy conditions or early cancer stages, and a tumor promotor in later cancer stages [16–18]. It can also lead to EMT, as well as ECM deposition and remodeling, resulting in fibrosis [9, 20].

These wide-ranging effects of TGF-β signaling can play an important role in the progression of numerous disease states, including both cancer and fibrosis. Given this, efforts have been made to target TGF-β signaling and block these detrimental downstream effects [142]. A number of methods have been employed to this end [69, 143, 144]. These approaches include the development of monoclonal antibodies that target TGF-β ligands [145]. By doing so, the TGF-β ligands can no longer bind to their receptors, in effect blocking the ligand-receptor interaction, and eliminating downstream signaling targets from being activated. The monoclonal antibodies under investigation include lerdelimumab (CAT-152) [146], metelimumab (CAT-192) [147], STX-100 [148], with the drug fresolimumab (GC1008) [149–152] showing the most promise, with ongoing enrollment in twelve clinical trials [153]. Other methods employed to block TGF-β signaling involve targeting the TGF-β receptor itself. By creating small molecules that inhibit the receptor’s kinase activity, the downstream signaling can effectively be attenuated. A few of the small molecules that are currently under investigation for this purpose are galunisertib (LY2157299) [154, 155] and SB431542 [156, 157].

One interesting method of targeting TGF-β signaling involves creating “decoy” receptors. These “decoy” receptors are composed of recombinant proteins that consist of the extracellular domain of TGF-β receptors fused to an immunoglobulin Fc domain. In effect, they sequester the TGF-β ligands which bind to the “decoys”, reducing the TGF-β ligands available for binding to the actual receptor, and thereby inhibiting downstream signaling of the pathway. These receptor “decoys” include the proteins sotatercept (ACE-011) [158–162] and luspatercept [163–165]. These studies have moved from the preclinical phase to the clinical phase, with 23 studies for sotatercept and 38 for luspatercept currently listed on ClinicalTrials.Gov at the time of writing. Another method utilizes antisense oligonucleotides. These are designed to target and degrade TGF-β ligand mRNA, which results in lower levels of TGF-β proteins being produced, decreasing the availability of ligand for receptor ligand binding, and decreasing downstream signaling. One example agent in this class of drugs is trabedersen, which has moved from preclinical to clinical trials, with 5 studies currently listed on ClinicalTrials.Gov [166].

**Fig. 2:**
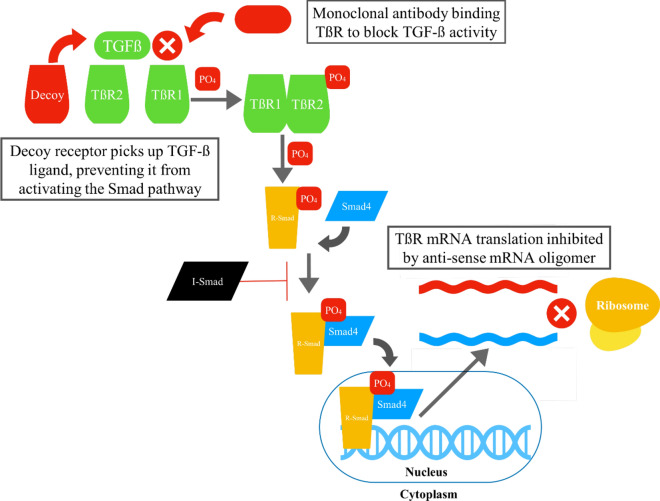
Graphical illustration of how developing therapies interact with TGF-β signaling. Multiple options are being explored. These include the decoy interception of the ligand itself, as well as a focus on targeting downstream signaling with anti-sense RNA inhibition of TßR RNA

It is important to note that given the wide-ranging actions of TGF-β, the development of any therapies targeting TGF-β signaling must proceed with caution. TGF-β plays many essential roles in normal physiology, the inhibition of which may cause significant clinical side effects [69]. The side effects include issues such as impaired wound healing, immune system dysregulation, cardiovascular toxicity, the formation of tumors, and GI disturbances [142, 167]. Care must be taken to develop therapies in a targeted and precise manner in order to avoid off target dose limiting side effects (Fig. 2).

#### Future directions of theranostic imaging and targeted treatment of TGF-β signaling

One important avenue of research in the field has been to develop better methods of imaging TGF-β signaling. As methods of imaging improve, we can hope to develop better targeted therapies for TGF-β signaling. In particular, the field of theranostics represents a potentially powerful new method to both visualize and treat pathologies [168, 169]. Theranostics is a field of medicine that combines diagnostics and therapeutic treatment to tailor personalized therapies for individual patients. Theranostic agents are designed to integrate diagnostic and therapeutic capabilities within a single platform, facilitating personalized medicine approaches [170–172]. Currently, this method has been most utilized in the field of oncology to visualize and treat tumors, however future applications need not be restricted to the field of oncology. Various strategies have been developed to visualize TGF-β signaling and its downstream targets, although these methods are primarily used for research purposes and have not yet been widely adopted as agents in the clinical setting [32, 169].

The direct imaging of TGF-β signaling using theragnostic agents is still in early stages. However, there has been recent research developing theragnostic agents that target TGF-β signaling pathways [169]. TGF-β ligands, inhibitors, or activators can be radiolabeled and injected into the subject. Using PET or single-photon emission computed tomography (SPECT), the binding of the radiolabeled TGF-β ligands or inhibitor can then be visualized in vivo*.* Studies have been investigating TGF-β activity using radiolabeled ligands in both cancer and fibrosis. In one study, 89-Zr was conjugated to fresolimumab for use in a hamster breast cancer model. Using PET imaging, the 89-Zr labeled fresolimumab was found to be highly correlated with tumor sites with ulceration and scar tissue, which is consistent with processes that TGF-β is active in. This result highlights the potential of in vivo TGF-β imaging and suggest the potential use of fresolimumab as an anti-cancer therapy [173]. Another strategy for in vivo TGF-β imaging has been using a 18-F-αvβ6. Integrin αvβ6 plays an important role in activating TGF-β. Integrin αvβ6 activates TGF-β by binding to its latency-associated peptide, triggering a conformational change that releases the active TGF-β for biological functions [174]. One group used a fluorine-18 radiolabeled αvβ6 radioligand in human non-small cell lung cancer patients who were recently treated with radiation therapy and healthy controls. Using PET, they found that in patients who were recently treated with radiation therapy had higher PET uptake of the radioactive ligand compared to the healthy controls. The results from this study show that the use of αvβ6-PET imaging may be useful in assessing for TGF-β generated fibrosis, especially in the setting post radiation therapy [175].

Another strategy in theragnostics is to use nanoparticles. Nanoparticles, tiny particles measuring between 1 and 100 nm, are utilized in theranostics for both diagnostic and therapeutic purposes. Nanoparticles are engineered to carry drugs or proteins and are designed to target specific cells or tissues. They can also carry imaging agents, allowing for real-time monitoring of drug delivery and treatment effectiveness, thereby integrating therapy and diagnostics into a single platform [176]. Nanoparticles can be functionalized with various modalities in order to act in vivo [177]. For example, one group used Poly(ethylene glycol)-poly(aspartic acid) (PEG–PAsp)-coated magnetite nanoparticles which are able to be tracked using non-invasive imaging modalities such as MRI. This group used a mouse model of pancreatic cancer, where they injected PEG-PAsp-coated magnetite nanoparticles along with a TGF-β inhibitor. They found that injection of the nanoparticles with the TGF-β inhibitor led to a significant increase in T2 imaging. This study highlights the potential for magnetite nanoparticles in improving diagnostic capacities for difficult to image and treat cancers, such as pancreatic adenocarcinoma [178]. Another group used iron oxide and ferulic acid co-encapsulated poly(lactic-co-glycolic acid) nanoparticles utilizing surface modification with cRGD peptides for integrin-targeted clinical PET-MRI traceable treatment of liver fibrosis. They found that by using these nanoparticles to target the TGF-β/Smad and NF-κB pathways, they were able to effectively both stage liver fibrosis as well as treat it by degrading collagen and modulating immune responses in their mouse model [179].

The recent advances that have been made in theragnostic agent development have the potential to facilitate personalized treatment strategies for diseases involving dysregulated TGF-β signaling. However, it is important to note that most of these approaches are still in the preclinical or early clinical stages of development, and further research is needed to optimize their safety, specificity, and efficacy before they can be widely adopted in clinical practice.

## Conclusions

In this review, we discussed the intricate relationship between TGF-β signaling in fibrosis and cancer progression. TGF-β, initially seemingly acting as a tumor suppressor, becomes a tumor promoter in pathological states. We present evidence that suggests its role in fibrosis is actually more accurately considered a pro-carcinogenic state. Fibrotic tissues, characterized by an excessive extracellular matrix, fosters an environment conducive to cancer development and resistance to therapies. The transition from promoting fibrosis to facilitating tumorigenesis underscores TGF-β's significance in disease progression and its potential as a therapeutic target for both fibrosis and cancer. The mechanisms of TGF-β signaling, from its involvement in ECM production and fibroblast activation in fibrosis, to its role in tumor microenvironment dynamics and cancer cell invasiveness, position it as a focal point for future therapeutic strategies.

## Data Availability

Not applicable.

## Abbreviations

AKT: Protein kinase B
BCL-xL: B-cell lymphoma-extra-large
BCL2: B-cell lymphoma 2
BI-RADS: Breast imaging reporting and data system
BMF: Bcl-2 modifying factor
BMP: Bone morphogenetic protein
Bim: Bcl-2 interacting mediator of cell death
CD87: Urokinase plasminogen activator receptor
CDK: Cyclin-dependent kinase
CIP1: Cyclin-dependent kinase interacting protein 1
CUG2: Cancer upregulated gene 2
DNA: Deoxyribonucleic acid
DPC4: Deleted in pancreatic cancer 4
ECM: Extracellular matrix
EGFR: Epidermal growth factor receptor
EMT: Epithelial-mesenchymal transition
ERK: Extracellular signal-regulated kinase
FAK: Focal adhesion kinase
FDA: Food and drug administration
G1: Growth 1 phase
GTPases: Guanosine triphosphatases
HER2: Human epidermal growth factor receptor 2
I-SMAD: Inhibitory SMAD
IPF: Idiopathic pulmonary fibrosis
JNK: C-Jun N-terminal kinase
KRAS: Kirsten rat sarcoma viral oncogene homolog
lncRNA: Long non-coding RNA
MAPK: Mitogen-activated protein kinase
miR: MicroRNA
MMPs: Matrix metalloproteinases
MRI: Magnetic resonance imaging
mTOR: Mechanistic target of rapamycin
NF-κB: Nuclear factor kappa-light-chain-enhancer of activated B cells
NSCLC: Non-small-cell lung cancer
Notch: Neurogenic locus notch homolog protein
P15: Cyclin-dependent kinase 4 inhibitor B
P21: Cyclin-dependent kinase inhibitor 1
p21WAF1: Cyclin-dependent kinase inhibitor 1A
PD-L1: Programmed death-ligand 1
PDAC: Pancreatic ductal adenocarcinoma
PDGF: Platelet-derived growth factor
PDGF-B: Platelet-derived growth factor subunit B
PEG–PAsp: Poly(ethylene glycol)-poly(aspartic acid)
PET: Positron emission tomography
PI3K: Phosphoinositide 3-kinase
R-SMAD: Receptor SMAD
SCLC: Small-cell lung cancer
SMAD: Small mothers against decapentaplegic
Sox: Sry-related HMG-box
Sp1: Specificity protein 1
SPECT: Single-photon emission computed tomography
TGF-β: Transforming growth factor-beta
TIMPs: Tissue inhibitors of metalloproteinases
TRAF: TNF receptor-associated factor
TβRI: TGF-β receptor type I
TβRII: TGF-β receptor type II
VEGF-D: Vascular endothelial growth factor D
Wnt: Wingless/Integrated
α-SMA: Alpha smooth muscle actin
